# Edward Jenner: A Beacon of Hope in the Age of Disease

**DOI:** 10.7759/cureus.69472

**Published:** 2024-09-15

**Authors:** Lavanya Balaji, Nandhini Ravella Venkatasubramanyam, Navin Umapathy, Subbalakshmi Easwaran, Neelusree Prabhakaran

**Affiliations:** 1 Department of Microbiology, Saveetha Medical College and Hospitals, Saveetha Institute of Medical and Technical Sciences, Saveetha University, Chennai, IND; 2 Department of Pediatrics, Saveetha Medical College and Hospitals, Saveetha Institute of Medical and Technical Sciences, Saveetha University, Chennai, IND

**Keywords:** edward jenner, historical vignette, small pox, smallpox variolation, vaccine

## Abstract

Edward Jenner, born in 1749 in Berkeley, Gloucestershire, England is widely recognized as the pioneer of the smallpox vaccine, a breakthrough that paved the way for the eventual eradication of the disease. This article traces Jenner's journey from his early education and apprenticeship under renowned surgeon John Hunter to his groundbreaking work on vaccination. Jenner's keen observations led him to hypothesize that cowpox could provide immunity against smallpox, which he confirmed through an experiment in 1796. Despite initial skepticism and the continued practice of variolation, Jenner's findings gained acceptance, and his work laid the foundation for modern immunology. The article also explores Jenner's personal life, his contributions to the medical community, and the eventual global impact of his work, culminating in the eradication of smallpox. Jenner's legacy is a testament to the power of scientific inquiry and its transformative effect on public health.

## Introduction and background

**Figure 1:**
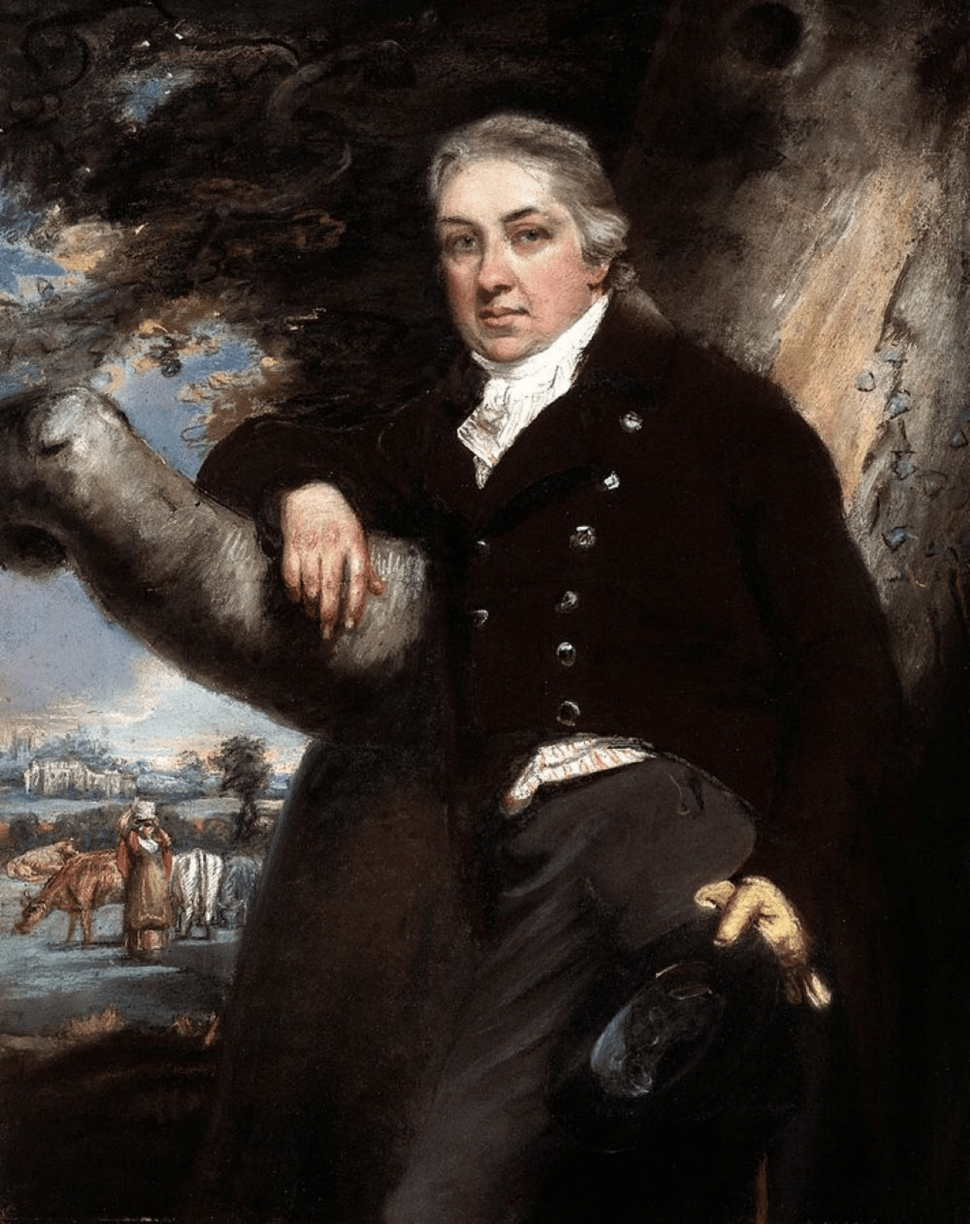
Picture of Edward Jenner Photo credits: John Raphael Smith, public domain, via Wikimedia Commons

Edward Jenner (Figure 1), born on 17 May 1749 in Berkeley, Gloucestershire, England, was the eighth of nine children. His father, Reverend Stephen Jenner, served as the vicar of Berkeley, ensuring that Edward received a solid foundation in education. Tragically, he was orphaned at the age of five, leading him to live with his older brother. During his formative years, Jenner attended Katherine Lady Berkeley's School in Wotton-under-Edge and later continued his studies in Cirencester. Jenner's early exposure to variolation, a form of smallpox inoculation, had a profound and lasting impact on his health, sparking his interest in medicine. At 14, he began a seven-year apprenticeship under Daniel Ludlow, a surgeon in Chipping Sodbury, where he gained invaluable experience [1]. In 1770, at the age of 21, Jenner furthered his medical education under the esteemed surgeon John Hunter at St. George's Hospital in London. Hunter, renowned as an eminent surgeon in England, was also highly regarded as a distinguished biologist, anatomist, and experimental scientist, whose profound influence greatly impacted Jenner [2]. The two years Jenner spent under Hunter’s mentorship further fueled his curiosity in natural science and solidified his approach to scientific discovery, embodied in Hunter's motto, "Don't think, try." Edward Jenner was elected a fellow of the Royal Society in 1788 primarily for his groundbreaking study on the behavior of the European cuckoo (*Cuculus canorus*). Under the mentorship of John Hunter, Jenner’s research focused on the cuckoo's unique practice of brood parasitism. He observed that cuckoos lay their eggs in the nests of smaller birds, such as reed warblers, and that the cuckoo chick instinctively ejects the host's eggs or chicks after hatching, ensuring it receives full care from the foster parents. Jenner's study significantly advanced the understanding of instinctual animal behavior, earning him recognition within the scientific community [3]. Jenner also made significant strides in clinical surgery during his time in London, including devising an improved method for preparing tartar emetic [3]. Hunter and Jenner maintained a close friendship and collaboration until Hunter's death in 1793. By 1773, he had returned to Berkeley, where he established himself as a successful family doctor and surgeon. After twenty years of practice, he earned an MD degree from the University of St. Andrews in 1792, cementing his reputation as a distinguished medical professional. Jenner was also a founding member of the Fleece Medical Society, later known as the Gloucestershire Medical Society, which held meetings in the parlour of the Fleece Inn, Rodborough. The society's members dined together and presented papers on various medical topics. Jenner contributed works on angina pectoris, ophthalmia, and cardiac valvular disease and also discussed cowpox.

## Review

The practice of variolation in the 18th century

In the 18th century, smallpox was a highly fatal disease, claiming approximately 10% of all lives, with a particularly devastating impact on infants and children. In villages, up to half of those infected would succumb to the disease, and survivors often bore disfiguring scars or suffered from blindness. It was widely known that individuals who survived smallpox gained immunity from future infections, leading to the practice of variolation. Prior to the discovery of vaccination, the most successful method for combatting smallpox was inoculation. The term "inoculation" originates from the Latin word "inoculare," which means "to graft." Inoculation entailed subcutaneously introducing the smallpox virus into nonimmune individuals using a lancet moistened with fresh matter from a mature pustule of a person with smallpox [2,4]. The substance was then inserted under the skin on the arms or legs of the nonimmune person. The terms inoculation and variolation were frequently used interchangeably. Variolation, also referred to as inoculation, is thought to have been practiced in Africa, India, and China long before the 18th century when it was brought to Europe [2,5].

**Figure 2:**
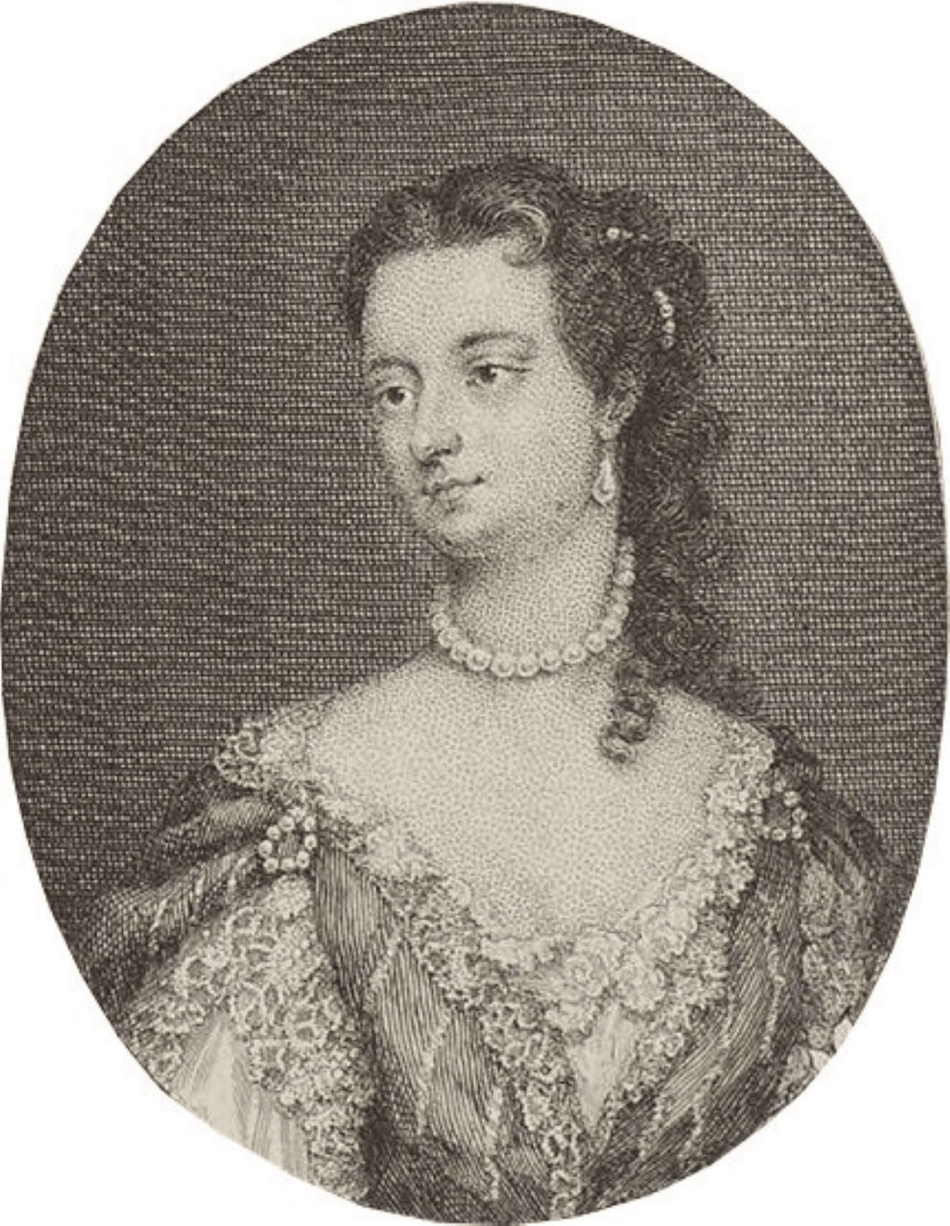
Picture of Lady Mary Wortley Montagu. Photo credits: George Vertue, public domain, via Wikimedia Commons

Lady Mary Wortley Montague (Figure 2), having experienced the devastating effects of smallpox and having lost her own brother to the disease, learned of variolation during her time in the Ottoman Empire in 1717. Eager to safeguard her children, she arranged for her son to be inoculated in Istanbul in 1718 and later facilitated her daughter's inoculation in London in 1721, sparking interest in the procedure within the royal court. This led to a significant trial in 1721, during which six prisoners were inoculated under the supervision of court physicians. Remarkably, all six prisoners survived and were subsequently found to be immune when later exposed to smallpox [6]. The success of these trials paved the way for the widespread adoption of variolation in Europe despite the associated risks of death, further outbreaks, or the transmission of other diseases such as tuberculosis and syphilis. The mortality rate from the procedure was 10 times lower than that of natural smallpox infections, making it a popular and widely accepted method of protection among both the nobility and the general population and ultimately laying the groundwork for the future development of vaccination.

In 1757, a significant event occurred in Gloucester, England. An eight-year-old boy was among the thousands who underwent smallpox inoculation that year. This procedure proved successful as the boy developed a mild case of the disease and gained immunity. Remarkably, this boy was none other than Edward Jenner, who would later become renowned for pioneering the smallpox vaccine [7].

Jenner's pioneering vaccination efforts and their initial reception

**Figure 3:**
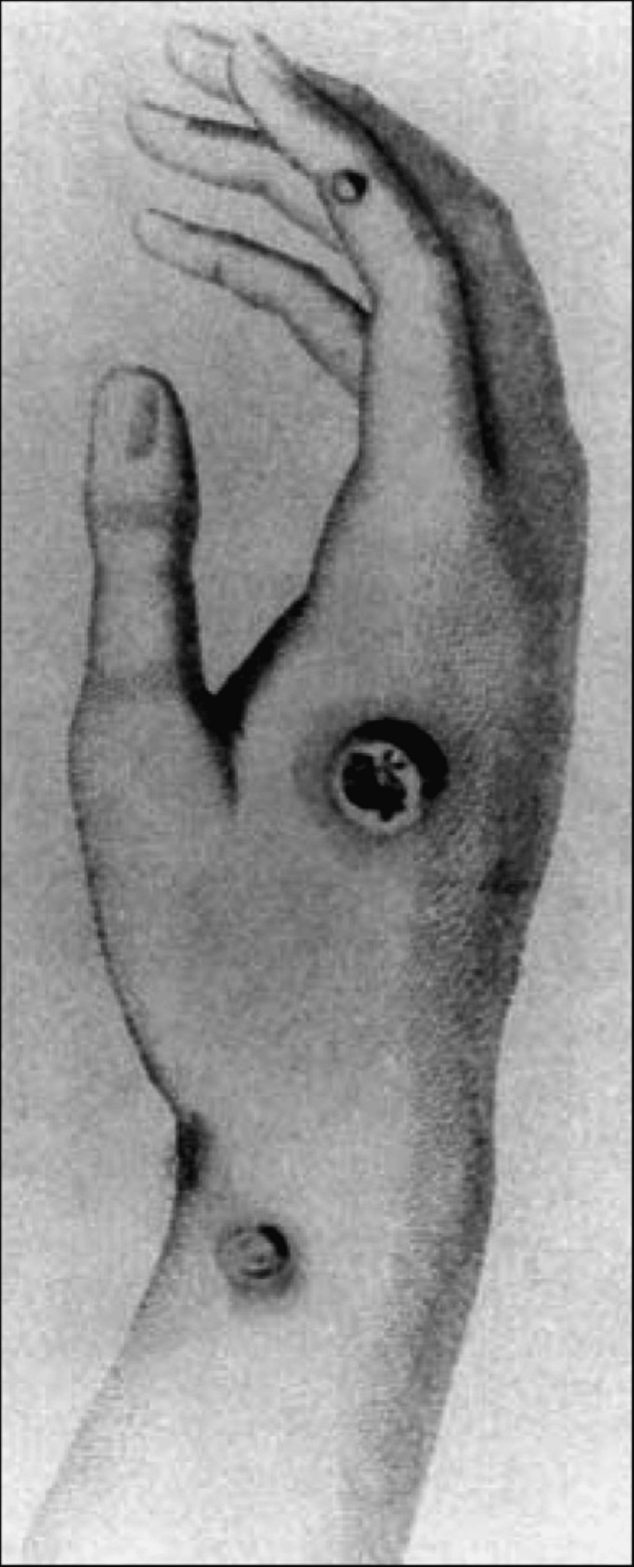
The hand of Sarah Nelms. Photo courtesy of the National Library of Medicine.

Edward Jenner's journey toward the discovery of the smallpox vaccine began during his apprenticeship with George Hardwicke, where his curiosity about the protective effects of cowpox first emerged. It wasn't until 1796, however, that he took a significant step in what would ultimately lead to the eradication of smallpox, one of humanity's most devastating diseases [1]. For years, Jenner had heard tales that dairymaids who contracted cowpox, a relatively mild disease, were somehow protected from the far deadlier smallpox. Reflecting on these stories, Jenner hypothesized that cowpox not only provided immunity against smallpox but could also be deliberately transmitted from one person to another to confer protection. In May 1796, Jenner encountered Sarah Nelms, a young dairymaid with fresh cowpox lesions on her hands and arms (Figure 3) [2,8].

**Figure 4:**
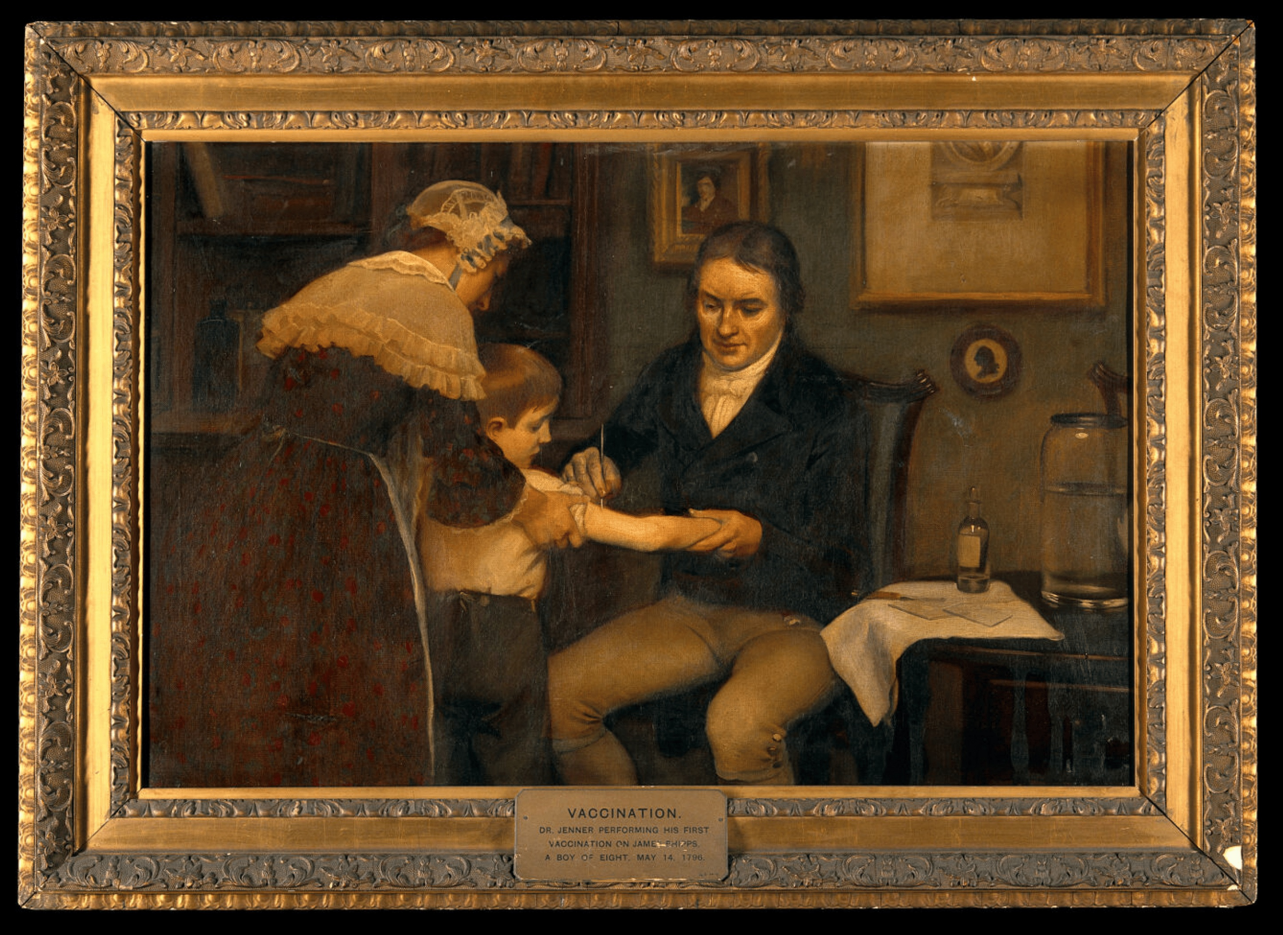
Dr Jenner performing his first vaccination, 1796. Oil painting by Ernest Board. Licence: Public Domain Mark Credit: Vaccination: "Dr Jenner performing his first vaccination, 1796". Oil painting by Ernest Board. Source: Wellcome Collection [9].

On May 14, 1796, Jenner took material from Nelms' lesions and used it to inoculate an eight-year-old boy named James Phipps (Figure 4). Over the next several days, Phipps developed mild symptoms such as fever and discomfort, but he quickly recovered. To test whether Phipps was now immune to smallpox, Jenner inoculated him again in July 1796, this time using material from a fresh smallpox lesion. Remarkably, Phipps did not develop the disease, leading Jenner to conclude that the boy was fully protected against smallpox. This groundbreaking experiment was initially rejected by the Royal Society in 1797, but Jenner persisted. In 1798, he privately published a booklet titled "An Inquiry into the Causes and Effects of the Variolae Vaccinae", where he detailed his findings [2,8]. Jenner's description of his first successful vaccination highlighted how he inoculated Phipps with cowpox material taken from Nelms' lesions. After observing Phipps' mild reaction, Jenner confirmed the boy's immunity to smallpox through subsequent inoculations with smallpox material, which had no effect. Jenner continued to promote his findings in subsequent publications, including "*Further Observations on the Variolae Vaccinae*" in 1799, where he detailed how to distinguish cowpox lesions from other similar pustular diseases [8]. 

In 1800, Jenner published "*A Continuation of Facts and Observations Relative to the Variolae Vaccinae or Cow Pox*", chronicling the success of his vaccination efforts as his colleagues spread the practice throughout their communities [10]. Despite the growing recognition of vaccination's life-saving potential, the practice of variolation-deliberately infecting people with smallpox to induce immunity-persisted in the UK. This allowed smallpox to continue circulating and causing periodic epidemics. Jenner's contributions were not fully recognized during his lifetime. Although vaccination became widely accepted, and Jenner sent cowpox material to those who requested it, variolation continued in the UK, and Jenner did not receive the recognition he deserved. It wasn't until 1821, after the death of King George III, that Jenner was appointed Physician Extraordinary to the new King, George IV. However, he was never knighted for his work.

Personal life

In March 1788, Edward Jenner married Catherine Kingscote, a woman he likely encountered during a balloon experiment. Jenner's test balloon is said to have descended into Kingscote Park, the Gloucestershire estate belonging to Catherine's father, Anthony Kingscote. The couple had three children: Edward Robert (1789-1810), Robert Fitzharding (1792-1854), and Catherine (1794-1833). The Jenner family resided in the Chantry House, which was established as the Jenner Museum in 1985. Jenner constructed a small hut in the garden called the "Temple of Vaccinia," where he provided free vaccinations to the underprivileged [2,11]. Between 1810 and 1815, Jenner endured the loss of his eldest son, Edward, his sisters Mary and Anne, and his wife, Catherine, all to tuberculosis. In 1820, he suffered a stroke but recovered. On January 23, 1823, after visiting his last patient, Jenner was found in his study, having suffered a massive stroke. He died on January 26, 1823, and was buried near the altar of Berkeley Church alongside his family.

Edward Jenner’s accolades and legacy

Edward Jenner was acclaimed for his pioneering contributions to natural history and medicine, which earned him election to the Royal Society in 1788. This honor recognized his exceptional research on the European cuckoo (Cuculus canorus), particularly his detailed study, “*Observations on the Natural History of the Cuckoo*”, which explored the cuckoo’s brood parasitism. Jenner also contributed to the understanding of zoology and botany, collecting specimens and exchanging knowledge with other eminent scientists of his time [3]. His keen observational skills and commitment to scientific inquiry helped expand the Royal Society's understanding of both medicine and natural history. A significant milestone in his career was marked by the conferral of an honorary Doctor of Medicine degree from the University of Oxford in 1807. In 1802, King George III bestowed royal recognition upon Jenner for his pioneering work on vaccination, extending support and admiration without officially knighting him. The enduring legacy of Jenner's work was acknowledged with the erection of a memorial in Berkeley, Gloucestershire, in 1858 known as the Jenner Memorial [10]. The Jenner Institute, established in 2005 at the University of Oxford, represents a lasting tribute to his contributions, focusing on vaccine development and advancements in immunology. Jenner's global impact can be seen in the eventual eradication of smallpox and the widespread adoption of vaccination as a standard practice in disease prevention. These accolades reflect the profound and lasting impact of Jenner's work on both medicine and public health [12].

Paving the legacy forward

Jenner persisted in advocating for vaccination until his demise in 1823 at the age of 74. It was not until 1840 that the UK Parliament formally prohibited variolation, thereby establishing cowpox vaccination as the authorized policy. Throughout the 19th and early 20th centuries, owing to the absence of methods for characterizing viruses, a varied assortment of vaccines was employed globally. Initially, the majority of cowpox vaccines were derived from Jenner's original material and transmitted from person to person. Over time, these sources intermingled with other isolates, including those obtained from cows and conceivably smallpox itself. By the conclusion of the 19th century, vaccines were being derived from calf skin subsequent to escarification [8]. In the United States, numerous isolates were in circulation, with some presumed to have originated from Jenner's material, although their specific provenance remained unknown. In due course, the vaccine employed in the United States as part of the worldwide smallpox eradication endeavor was designated Vaccinia, produced from infected calf skin by Wyeth under the designation Dryvax® [8,12]. Jenner's innovative undertakings laid the groundwork for this global initiative, ultimately culminating in the eradication of smallpox.

## Conclusions

Edward Jenner's pioneering work on the smallpox vaccine stands as one of the most significant milestones in medical history. Through his observations and experiments, Jenner laid the groundwork for the field of immunology and demonstrated the potential of vaccination to combat infectious diseases. His meticulous approach, despite initial skepticism from the medical community, eventually led to the widespread adoption of vaccination, which played a crucial role in the eventual eradication of smallpox. Jenner's contributions extended beyond the development of the vaccine itself; he also established principles that would guide future research in disease prevention and immunization. His legacy is a testament to the power of scientific inquiry and its capacity to bring about transformative change in public health.
